# Prospective effect of linkers type on the anticancer activity of pemetrexed-monoclonal antibody (atezolizumab) conjugates

**DOI:** 10.12688/f1000research.140284.2

**Published:** 2024-03-11

**Authors:** Faten Q. Ibraheem, Nidhal K. Maraie, Basma Talib Al-Sudani, Ayad M.R. Raauf

**Affiliations:** 1 pharmaceutics, Mustansiriyah University, Baghdad, Baghdad Governorate, 10011, Iraq; 2 pharmaceutics, Al-Farahidi University, Baghdad, Baghdad Governorate, 10011, Iraq; 3 pharmacology, Mustansiriyah University, Baghdad, Baghdad Governorate, 10011, Iraq; 4 pharmaceutical chemistry, Al-Farahidi University, Baghdad, Baghdad Governorate, 10011, Iraq

**Keywords:** monoclonal antibody, antibody drug conjugate, pemetrexed

## Abstract

**Background:**

Conventional chemotherapy results in severe toxic side effects due to affecting normal and cancer cells. The conjugation of chemotherapy with mAb will improve the chemotherapy selectivity towards cancer cells and at the same time will potentiate immune system to detect and kill cancer cells. The aim of the study was to prepare atezolizumab-pemetrexed conjugate using two types of linkers (linker conjugated with -NH2 of lysine amino acid in the mAb).

**Methods:**

This study utilizes (for the first time) the mAb atezolizumab (AtZ) to prepare a new, selective conjugate carrier for pemetrexed (PMX) by using gamma amino butyric acid (GABA) as linker for the first time in comparison to the commonly used linker polyethylene glycol (PEG) using carbodiimide (EDC) / N-hydroxysulfosuccinimide (Sulfo-NHS) zero length cross linker. Stepwise evaluation for PMX-linkers linkage as well as mAb conjugates was evaluated by FTIR, ^1^HNMR, DSC, LC-MS, gel-electrophoresis as well as the anticancer activity against lung cells A549.

**Results:**

The work revealed that two molecules of GABA combined with PMX, which in turn conjugated with an average ratio of 4:1 with mAb, while one molecule of PEG combined with PMX, which in turn conjugated with mAb in the same average ratio. The IC _50_ for the prepared PMX-GABA-AtZ conjugate was 0.048 µM, which was much lower than PMX alone, antibody AtZ alone as well as PMX-PEG-AtZ conjugate in a dose and time dependent manner.

**Conclusions:**

The potential use of such conjugate that selectively directed to the overexpressed lung cells antigen in a low dose leading to reduction of serious side effects of PMX and the cost of therapeutically AtZ mAb used.

## Introduction

The beneficial effects of therapeutic monoclonal antibodies (mAbs) that are represented by selectivity, minimum toxicity, and immune system activation gave chance for protein in biotechnology, such as bispecific mAbs, antibody drug conjugate (ADC). The mAb developed for many indications, including cancer, autoimmune disorder, and infectious diseases. ^1^^,^^2^ Each mAbs has high affinity to specific antigen (overexpressed for example in diseased cancer cells), mAb applied as a carrier for drug through chemical conjugation to ensuring minimal drug loss during the transit to the target site, protect the drug from metabolism and premature clearance, and retain the drug at the target site. ^3^ ADCs consist of a monoclonal antibody, cytotoxic drug, and a linker to conjugate the drug with mAb. ^4^ The mAb is a promising target in anticancer field therapy that offers other advantage of improving exhausted immune cells (T cells) capability to detect and destroy tumor. ^5^^,^^6^ The interaction between the surface programmed death-1 (PD1) with its ligand (PDL1), which are expressed on the surface of T cells and tumor cells, respectively prevents immune mediated cancer killing. To enhance T-cells capability against cancer cells, several antibodies have been developed. ^7^^,^^8^ Atezolizumab (AtZ) was approved by the US FDA in 2016 for urothelial and metastatic lung cancer, gained approval for the treatment of advanced bladder cancer in 2017. ^9^^,^^10^ AtZ immunologically interrupts PDL1–PD1 binding and therefore prevents T cell exhaustion. ^11^ Pemetrexed (PMX) disrupts cellular replication by directly incorporating into the DNA, ^12^ approved by the FDA for treatment of advanced lung cancer ^13^^,^^14^ but unfortunately the physicochemical characteristics act as a barrier in its pharmacokinetic, where PMX diacid is practically insoluble in water, ^15^ while the PMX disodium salt fails in achieving high stability upon storage. ^16^ The most important points associated with PMX are lack of selectivity by affecting normal and cancer cells and serious side effects such as hepatic and hematological toxicities. ^17^ The aim of this work is to improve the selectivity and targeting of PMX towards lung cancer through conjugation with therapeutic mAb (AtZ) where both act for treatment of lung cancer cells and study the efficacy of conjugate using a new linker gamma amino butyric acid (GABA) in the conjugation in comparison to the commonly used linker polyethylene glycol (PEG). Such conjugation might reduce the serious side effects of PMX chemotherapy and reduce the high cost of using therapeutic mAb alone.

## Methods

### Materials

Atezolizumab (Tecentriq ^®^, F. Hoffmann-La Roche Ltd), pemetrexed diacid (purity 98%) with MW 427.41 g/mol, Hydroxy-2,5-dioxopyrrolidine-3-sulfonicacid sodium salt (Sulfo-NHS), EDC (1-ethyl-3-(3-dimethylaminopropyl) carbodiimide hydrochloride), all purchased from Baoji Guokang Bio-Technology Co., Limited. Amine-PEG _4_-COOH (α-amine-ω-propionic acid tetraethylene glycol) MW 265 g/mol, gamma-Aminobutyric acid (GABA) MW 103.12 g/mol purchased from Tunchem Pharm (Shanghai) Tech Co., Ltd. N, N-Dimethylformamide (<0.1% H _2_O) purchased from Sinopharm Chemical Reagent Co., Ltd. Thermo Scientific Slide -A- Lyzer Dialysis Cassettes 10K MWCOs., Thermo Scientific Zeba Spin Desalting Columns 40K MWCOs.

### Purification and lyophilization of the received marketed monoclonal antibody (Atezolizumab)

Dialysis was done to remove all the excipients included in the marketed AtZ mAb. The dialysis done according to Thermo Fischer scientific protocol for protein dialysis, ^18^ using Slide-A-Lyzer ^®^ Dialysis Cassettes, 10K MWCO (Thermo scientific, Prod # 66810, Lot # 10001172, U.S.A.). A total of 20 ml from the supplied AtZ solution were withdrawn under sterile aseptic laminar flow cabinet and divided into two dialysis cassettes, each cassette immersed in 1 L of sterile phosphate buffer saline (PBS) pH 7.6 and left stirring using magnetic stirrer (Misung scientific, MS-300HS, Korea) at 50 rpm and 2-8°C for 4 hr, the buffer was replaced twice in each 2 hr with fresh 1L PBS pH 7.6 medium. After 4 hr of stirring, the medium was replaced with 1 L fresh one and left overnight in 2-8°C. ^18^ The dialyzed antibody was transferred into an empty vial containing 4% (W/V) mannitol and 1% (W/V) sucrose then lyophilized. ^19^ The lyophilization process was done for 8 hr (-55°C and 0.07 mbar) using a CHRIST lyophilizer (ALPHA 1-2 LD plus, Germany) and the obtained lyophilized powder stored at 2 to 8°C for later use.

### Synthesis of pemetrexed-atezolizumab (PMX-AtZ) conjugate

**Figure 1.**
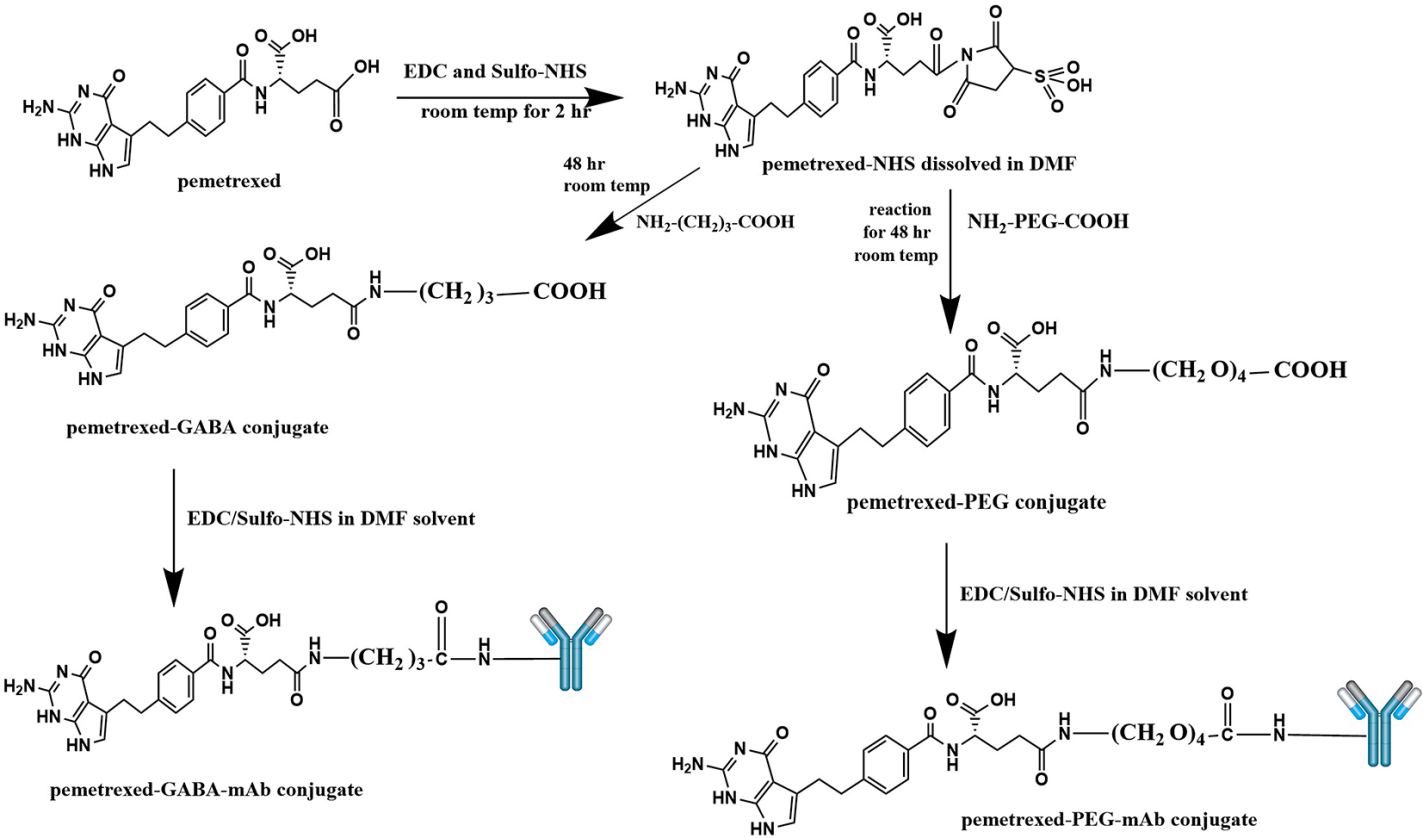
Suggested schematic illustration for the synthesis of pemetrexed linker conjugates and pemetrexed-linker-mAb conjugates. This figure is an original figure produced by the authors for this article.

The conjugation of PMX with AtZ mAb was done using two types of linkers (each one separately), the α-amine-ω-propionic acid tetraethylene glycol, which is NH _2_-PEG _4_-COOH and gamma-Aminobutyric acid (GABA). The procedure involved two steps: first; chemical conjugation of PMX with each linker to obtain PMX-linker conjugate and second; reacting the developed PMX-linker conjugate with the monoclonal antibody (AtZ), as shown in Figure 1.

Synthesis of PMX-linker conjugate (PMX linkage), the procedure involved activation of the carboxylic acid (carboxylic acids in general are not reactive enough to undergo nucleophilic addition directly) moiety in PMX using 1-ethyl-3-(3-dimethylaminopropyl) carbodiimide hydrochloride (EDC) and Hydroxy -2, 5-dioxopyrrolidine-3-sulfonicacid sodium salt (Sulfo-NHS) through the NHS ester formation, followed by reaction with a primary amine (in the linker molecule) to form an amide product.

For synthesis of PMX linkage with NH _2_-PEG _4_-COOH linker, PMX (0.04 mmol, 0.017 gm) was dissolved in 5 mL of dimethyl formamide (DMF) solvent, then EDC (0.06 mmol, 11 μl) and Sulfo-NHS (0.06 mmol, 0.013 gm) were added to pemetrexed solution in DMF. The solution was stirred by magnetic stirrer (Misung scientific, MS-300HS, Korea) at room temperature for 2 hr. Then NH _2_-PEG _4_-COOH (0.04 mmol, 0.010 gm) was added, and the mixture was stirred at room temperature for 2 days. The resultant solution was dialyzed in a dialysis bag (MWCO 3.5 kDa) against deionized water for 24 hrs with replacing the deionized water every 2 hr. Then, the obtained solution was freeze dried at (-55°C and 0.07 mbar) using a CHRIST lyophilizer (ALPHA 1-2 LD plus, Germany) to obtain the powder form of the PMX-NH _2_-PEG _4_-COOH linkage (symbol as PMX-PEG). ^16^^,^^20^ This procedure was repeated to link PMX with other linker (gamma amino butyric acid; GABA) using (0.04 mmol, 0.00412 gm) of GABA, to get PMX-GABA linkage as a powder.

For conjugation of PMX linkage with AtZ mAb, synthesis done by taking 17 mg of each of the obtained product separately (dried PMX-PEG and PMX-GABA linkages) and placed in 5 ml DMF solution containing mixture of 9 μl EDC/0.01 gm Sulfo-NHS and stirred by magnetic stirrer at room temperature for 2 hr, then 17 mg of AtZ mAb was added and stirring continued gently at 6°C for 48 hr using magnetic stirrer. The resultant mixture purified by desalting column (Zeba ^TM^ Spin Desalting Columns, 40K MWCOs, 10 ml, Thermo scientific, REF 87772, LOT XG361556, U.S.A.) ^21^ and the obtained products was freeze dried after adding 5% (W/V) mannitol: sucrose mixture (4:1), using a CHRIST lyophilizer.

### Characterization of AtZ-PMX conjugate

The characterization of the entire conjugate (AtZ-PMX) was carried out step by step. First step is characterization of PMX with linkers and this include:

1 Fourier transform infra-red (FTIR) analysis
2 Differential scanning calorimetric (DSC) analysis
3 Mass spectroscopy (MS) analysis
4 Proton nuclear magnetic resonance ( ^1^HNMR) analysis

The second step is characterization of (PMX-linker-AtZ) which symbolled (AtZ-PMX) and this include:

1 Gel electrophoresis
2 Mass spectroscopy

**Characterization of conjugation of PMX with linkers**

The characterization of linkage of PMX with NH _2_-PEG-COOH and PMX with GABA linker involved the following:

### Fourier transform infra-red (FTIR) analysis

The FTIR (Shimadzu 8400 S, Japan) was done using potassium bromide disc, wavelength (4000–500 cm ^-1^) was used for PMX-PEG linkage, PMX-GABA linkage, pure PMX, pure PEG, pure GABA, physical mixture of PMX and PEG, and the physical mixture of PMX with GABA. ^22^

### Differential scanning calorimetric (DSC) analysis

The DSC analysis (DSC 131EVO, France) was done for pure PMX, PEG, GABA, PMX-PEG linkage, and PMX-GABA linkage, using aluminum containers containing 2 mg of each sample, under nitrogen atmosphere 50 mL per minute, and a heat rate of 5°C in minute in temperature of 25-300°C. ^23^

### Mass spectroscopy (MS) analysis

Compounds screening was done using Bruker Daltonik (Bremen, Germany) Impact II ESI-Q-TOF System by direct injection. Stock Solution for each sample (pure PMX, pure GABA, pure PEG, PMX-PEG linkage, and PMX-GABA linkage) was prepared by dissolving 1 mg of each compound in 700 μl MeOH and 300 μl water, 1 μL were injected to get appropriate signal in mass spectrometer, ^24^ separation was done by UHPLC column (100 mm x 2.1 mm x 2.0 μm) Bruker solo2.0-C-18, flow rate of 0.51 mL in minute and temperature of 40°C.

### Proton nuclear magnetic resonance ( ^1^HNMR) analysis

The ^1^HNMR was done for PMX, PEG, GABA, PMX-PEG linkage, and PMX-GABA linkage using Bruker (BioSpin GmbH 500 MHz, Germany). ^1^H-NMR analysis performed using DMSO solvent and chemical shifts (δ) stated in parts per million. ^25^

### Characterization of PMX-linker-AtZ mAb conjugation

The prepared PMX-AtZ conjugate using either PEG or GABA as a linker were characterized by:

***Gel electrophoresis***

The SDS PAGE is a characterization tool for pure mAb, and the two PMX-AtZ mAb conjugates. SDS-PAGE gel assay kit (Elabscience ^®^, U.S.A., Cat No. E-IR-R305) was used for gel preparation. The vertical electrophoresis system (Bio-rad, PowerPac ^TM^ Basic, Singapore) was used for gel electrophoresis.

The separating gel concentration used was 6% (for protein with MW 50-150 KDa), and was prepared as follows: water, 30% Acrylamide/Bis-acrylamide solution, and Separating Gel Mix, were mixed in a clean dry beaker. Then 10% Ammonium Persulfate, and Tetramethylethylenediamine were added, mixed gently to avoid bubbles, the prepared mixture poured immediately into a clean assembled gel mold and kept until solidified for approximately 30-60 minutes then ethanol (1-2 ml) was added to flatten the gel surface. Ethanol was removed after gel solidified and any residual liquid in the assembled gel mold was removed gently by using absorbent paper.

The 5% stacking gel was prepared by gently mixing in dry, and clean beaker the following components: water, 30% Acrylamide/Bis-acrylamide solution, and Stacking Gel Mix. Then 10% Ammonium Persulfate and Tetramethylethylenediamine were added and poured immediately into the previously prepared solidified separating gel. Comb teeth were inserted, and gel kept until solidified for 30-40 minutes. ^26^^,^^27^ The solidified gel cassette fixed inside the electrophoresis tank; comb removed carefully. The electrophoresis tank filled with 1X Tris-Glycine buffer (Elabscience ^®^, U.S.A.).

Samples for gel electrophoresis (AtZ mAb, PMX-PEG-AtZ conjugate, and PMX-GABA-AtZ conjugate) were prepared by mixing 20 μl of each sample (concentration 0.5mg/ml) with 5 μl loading buffer (5X SDS Loading Buffer, Elabscience ^®^, U.S.A. Cat No. E-BC-R288), samples mixed with (vortex mixer, Faithfull, model MX-S). The prepared samples were heated (dry block heater, jSR, model JSBL-01T, korea) at 95°C for 5 minutes (heating speed up protein denaturation by increasing sample molecular motion), then samples were cooled to room temperature before loading.

A total of 10 μL of each sample loaded into the gel well (lane), 5 μL marker (pre-stained protein marker 10-180 KDa, Elabscience ^®^, U.S.A. Cat No. E-BC-R273) was loaded into gel lane. In another gel mold, samples without heating (keeping the temperature 20-25°C to avoid denaturation) were loaded into the gel lane.

The electrophoresis system was run at voltage 120V for about 2-3 hours. At the end of experiment the gel was removed from the tank and transferred directly into 20 mL staining solution (mixture of 2 g Coomassie Brilliant Blue R-250, 300 ml methanol, 100 ml glacial acetic acid, and 600 ml distilled water) and gel left gently shaking (Human, HumaRock, Germany) for approximately 1 hr. After 1 hr the gel transferred into the de-staining solution (mixture of 300 ml methanol, 100 ml glacial acetic acid, and 600 ml distilled water) and left shaking gently overnight. The gel washed with distilled water and band visualized by using Bio-Rad Chemidoc XRS Gel Imaging System (RRID:SCR_019690), model No. Universal Hood II, U.S.A.

### Mass spectroscopy

A Bruker (Bremen, Germany) was used for screening conjugates compounds. Stock Solution for each sample (unconjugated atezolizumab mAb, pemetrexed-PEG-mAb conjugate, and pemetrexed-GABA-mAb conjugate) was prepared by dissolving 7.25 mg in 50 ml ACN: water (1:1) and 0.1 formic acid, 125 ppm of sample injected (sample concentration) to get appropriate signal in mass spectrometer. The separation was performed using Bruker solo2.0-C18 column (100 mm x 2.1 mm), flow rate 0.3 mL/min, column temp of 60°C, and dry gas temp 300°C. ^28^ The mobile phases used were water with 0.1% formic acid (A) and acetonitrile with 0.1% formic acid (B), and the run was in gradient profile as follow: from 0-15 min (95% of A and 5% B), from 15-25min (10% A and 90% B), and from 25-30 min (95% A and 5% B).

### Cytotoxic activity study for the prepared pemetrexed-atezolizumab conjugates

Cytotoxic activity of PMX-AtZ conjugates using two types of linkers PEG and GABA (each one separately) were studied using lung cancer cells (A549) in comparison to pure PMX, pure AtZ, PMX-PEG linkage, and PMX-GABA linkage. Lung cancer cell line (A549) was purchased from American Type Culture Collection ATCC (Middlesex, UK) and stored in the Cell Bank of the Biomedical Research Centre at Mustansiriyah university.

### Viability and inhibitory concentration (IC _50_) of cells by MTT assay

The viability of A549 cancer cells after exposure to various samples, including pure PMX, PMX-PEG linkage, PMX-GABA linkage, pure mAb, PMX-PEG-AtZ conjugate, and PMX-GABA-AtZ conjugate was determined by MTT assay.

The A549 cell suspensions were dispensed into 96-well flat plates in a concentration of 5 x 10 ^3^ cells per well, incubated for 1 day under standard conditions; 4 x 10 ^3^ cells per well, incubated for 2 days; and 3 x 10 ^3^ cells per well, incubated for 3 days under standard conditions. ^29^^,^^30^ Cells were treated with same concentration range 0.05-100 μM of each sample, control group with only growth medium included within plate and plate incubated. After a 24 h, 48 h, and 72 h incubation, the cell culture medium was removed (after each recovery period) and incubated with MTT solution (3-(4,5-Dimethyl thiazol-2-yl)-2,5 Diphenyl tetrazolium Bromide), the medium was removed and DMSO was added to plates (remained at room temperature in the dark for fifteen minutes). The absorbance was measured at 540 nm with background absorbance correction at a wavelength of 650 nm, optical density of the control wells was used to determine the cells viability. ^31^^,^^32^ The dose response plotted over log concentrations and IC _50_ values calculated using Graph Pad Prism version 8.4.3 (RRID:SCR_002798), results represented standard error of the mean for triplicate data. ^33^ R is an open source alternative software which could be used for this.

## Results and Discussion

Two types of PMX-AtZ conjugates were prepared using two types of linkers (each one separately) including PEG (the commonly used linker) and GABA, which was used for the first time in drug-antibody conjugation in this work. Both conjugates were characterized and evaluated.

### FTIR characterization

**Figure 2.**
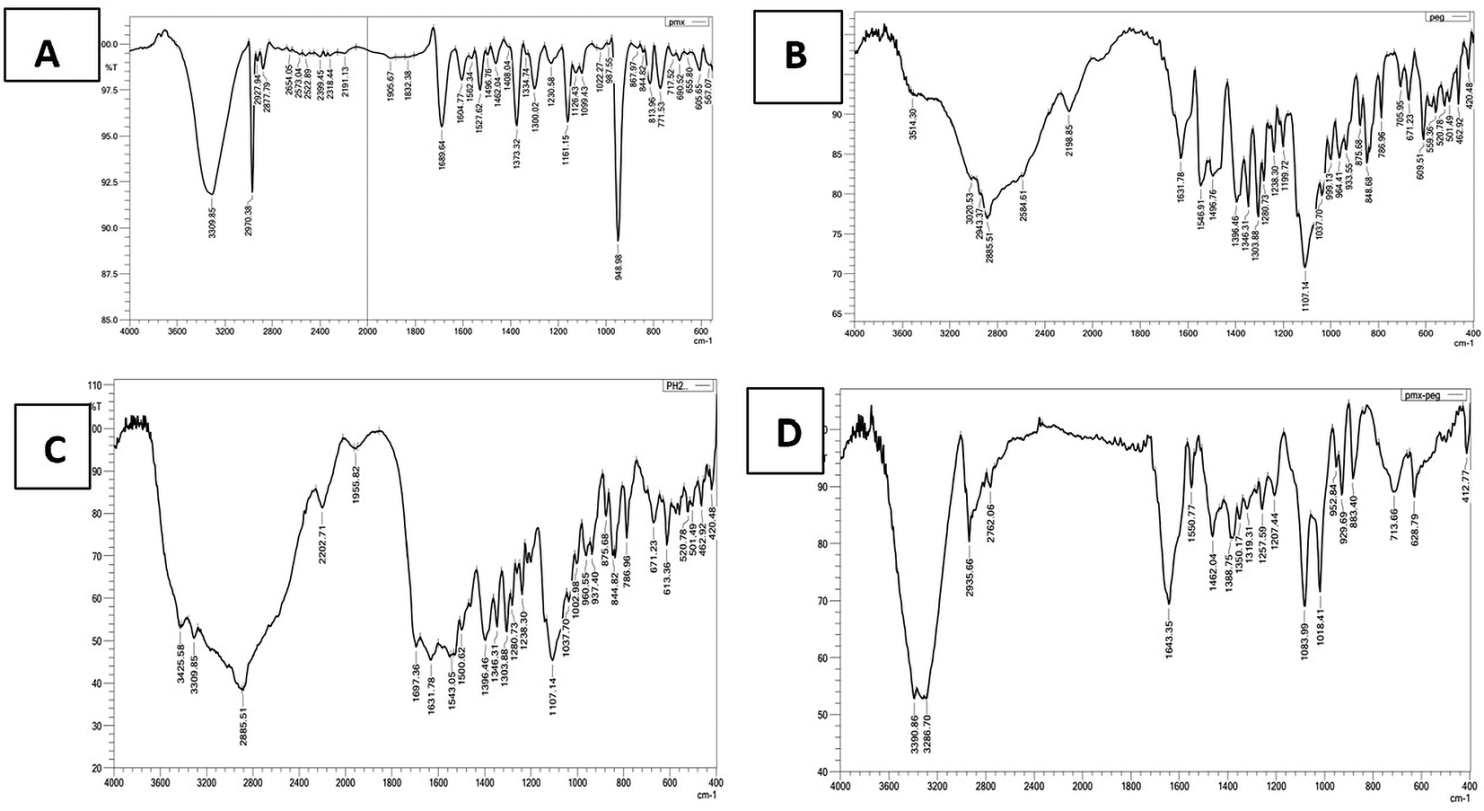
FTIR for (A) pure PMX, (B) PEG, (C) physical mixture (PMX and PEG), and (D) PMX-PEG linkage.

The FTIR spectrum ( Figure 2) of pure PMX displaced the characteristic stretching band O-H of COOH near 3400 cm ^-1^, stretching NH _2_ at 3309.85 cm ^-1^, stretching NH at 2970.38 cm ^-1^, stretching C-H aliphatic at 2877.79 cm ^-1^, C=O of COOH at 1689 cm ^-1^, C=C aromatic at 1527.62 cm ^-1^, and all these peaks similar to that reported. ^29^

The α-amine-ω-propionic acid tetra ethylene glycol (NH _2_-PEG _4_-COOH) displaced characteristics stretching C=O of COOH at 1631.78 cm ^-1^, stretching band O-H of COOH at 3514.30 cm ^-1^. ^34^ The mixture of PMX and PEG showed the same characteristics peaks observed in each ingredient separately with observable broadening in C=O of COOH at 1631.78 and 1697.36 cm ^-1^, which are for C=O of COOH of the PEG and PMX, respectively. The spectrum of the chemically linked PMX-PEG displaced a new sharp peak at 1643.35 cm ^-1^ and this not found neither in the physical mixture, nor in each molecule separately indicating chemical linkage and related to C=O of intended amide.

**Figure 3.**
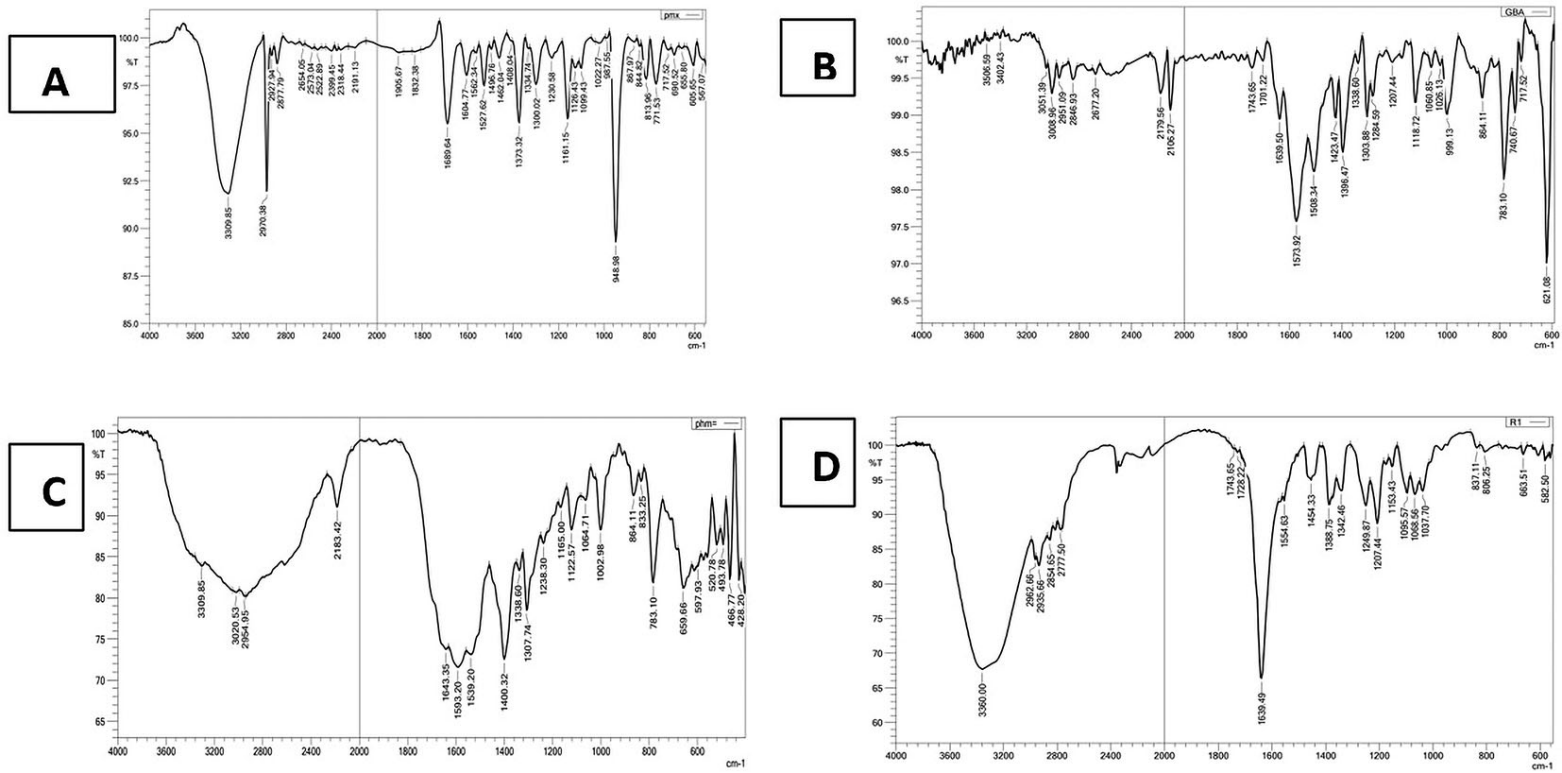
FTIR for (A) pure PMX, (B) GABA, (C) physical mixture (PMX and GABA), and (D) PMX-GABA linkage.

GABA ( Figure 3) showed aliphatic -CH peaks at 2951.09 cm ^-1^, 3008.96 cm ^-1^ and 2846.93 cm ^-1^. There were also bands corresponding to asymmetrical and symmetrical stretching vibration of carboxylate group at 1573.92 cm ^-1^ and 1396.47 cm ^-1^, respectively, stretching band O-H of COOH 3402.43 cm ^-1^ and 3506.59 cm ^-1^. The GABA FTIR is in accordance with reported data. ^35^ The physical mix of PMX and GABA showed the same characteristics peaks observed in GABA at 1338.6 and 783.1 cm ^-1^ and others related to PMX at 3309.85 cm ^-1^ and this appeared as a broad band with aliphatic -CH peaks of GABA. The chemically linked PMX-GABA displayed amide -NH stretching at 3360 cm ^-1^, sharp intense peak at 1639.49 cm ^-1^ related to C=O stretching of conjugated amide. The underlying data are available in a data repository. ^36^

### Characterization by DSC

**Figure 4.**
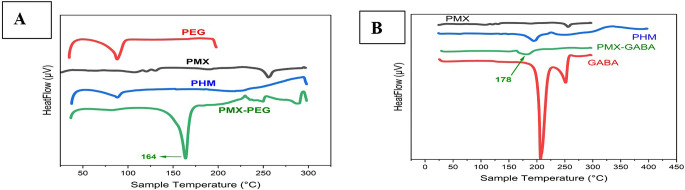
(A) DSC for PEG, PMX, PHM (physical mixture of PMX and PEG), PMX-PEG (pemetrexed-PEG linkage). (B) DSC for GABA, PMX, PHM (physical mixture of PMX and GABA), PMX-GABA (pemetrexed-GABA linkage).

Characterization by DSC done for PMX, PEG, GABA, physical mix in equal amount of PMX and PEG, physical mix in equal amount of PMX and GABA, PMX-PEG linkage, and PMX-GABA linkage. The DSC results ( Figure 4A) showed that PMX had a sharp narrow, intense endothermic peak at 252°C that represents melting point of the drug. ^15^ PEG spectrum displayed a strong endothermic peak at 88°C. ^37^ The physical mix of PMX and PEG displayed the two characteristics endothermic peaks of them, while the chemically linked one (PMX-PEG) displayed one new sharp intense endothermic peak at 164°C. GABA spectrum ( Figure 4B) showed an intense endothermic peak at 205°C in accordance with the melting point of GABA. ^38^ The physical mix of PMX and GABA displayed the two characteristics endothermic peaks, while PMX-GABA linkage spectrum had a new peak at 180°C, which is neither related to PMX nor to GABA, suggesting chemical linkage between drug and GABA linker. The underlying data are available in a data repository. ^39^

### Characterization by LC-MS

**Figure 5.**
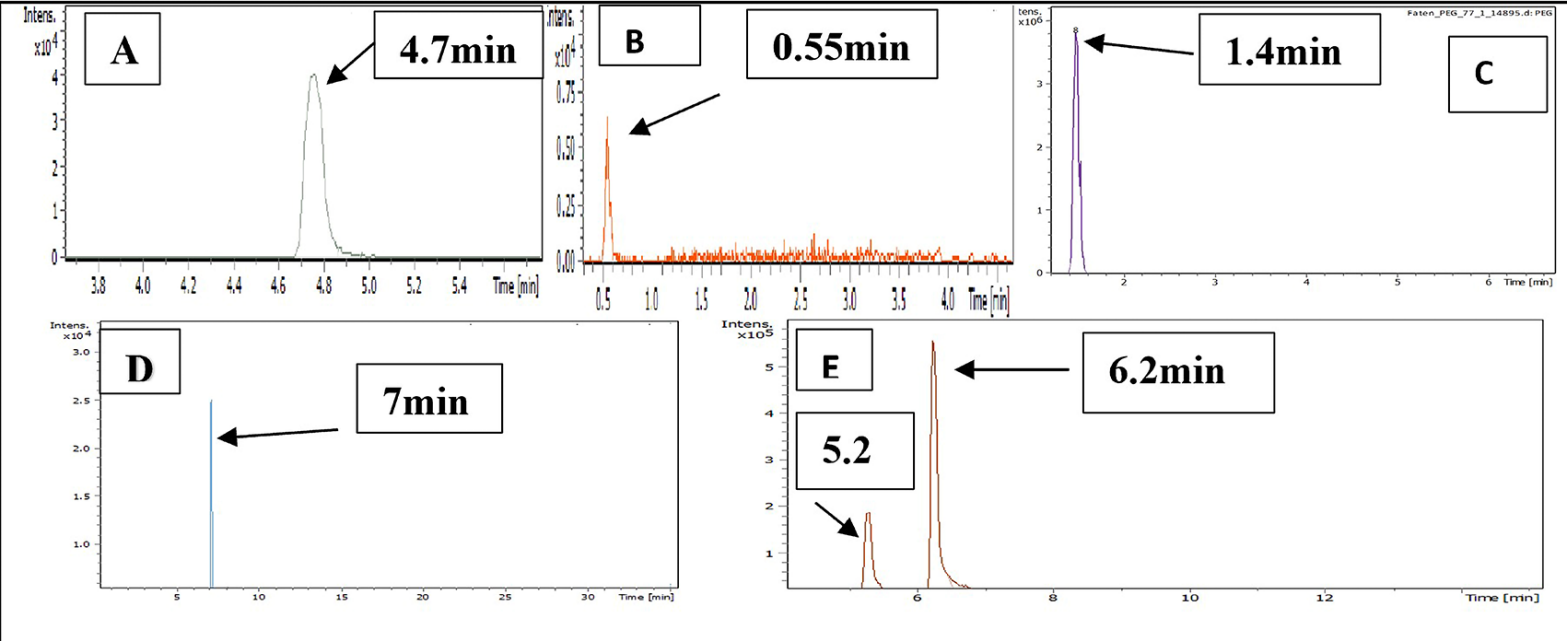
LC-MS chromatogram revealed the retention time of (A) pure PMX, (B) pure GABA, (C) pure PEG, (D) PMX-PEG linkage, and (E) PMX-GABA linkage.

LC-MS chromatograms of PMX, GABA, PEG, PMX-PEG linkage, PMX-GABA linkage is demonstrated in Figure 5, where the retention time for PMX at 4.7 min, GABA 0.55 min, PEG 1.4 min, PMX-PEG linkage 7 min, PMX-GABA linkage 5.2 and 6.2 min. It was obvious that the newly conjugated molecule (PMX-PEG and PMX-GABA) displayed different retention time of their pure molecules alone.

**Figure 6.**
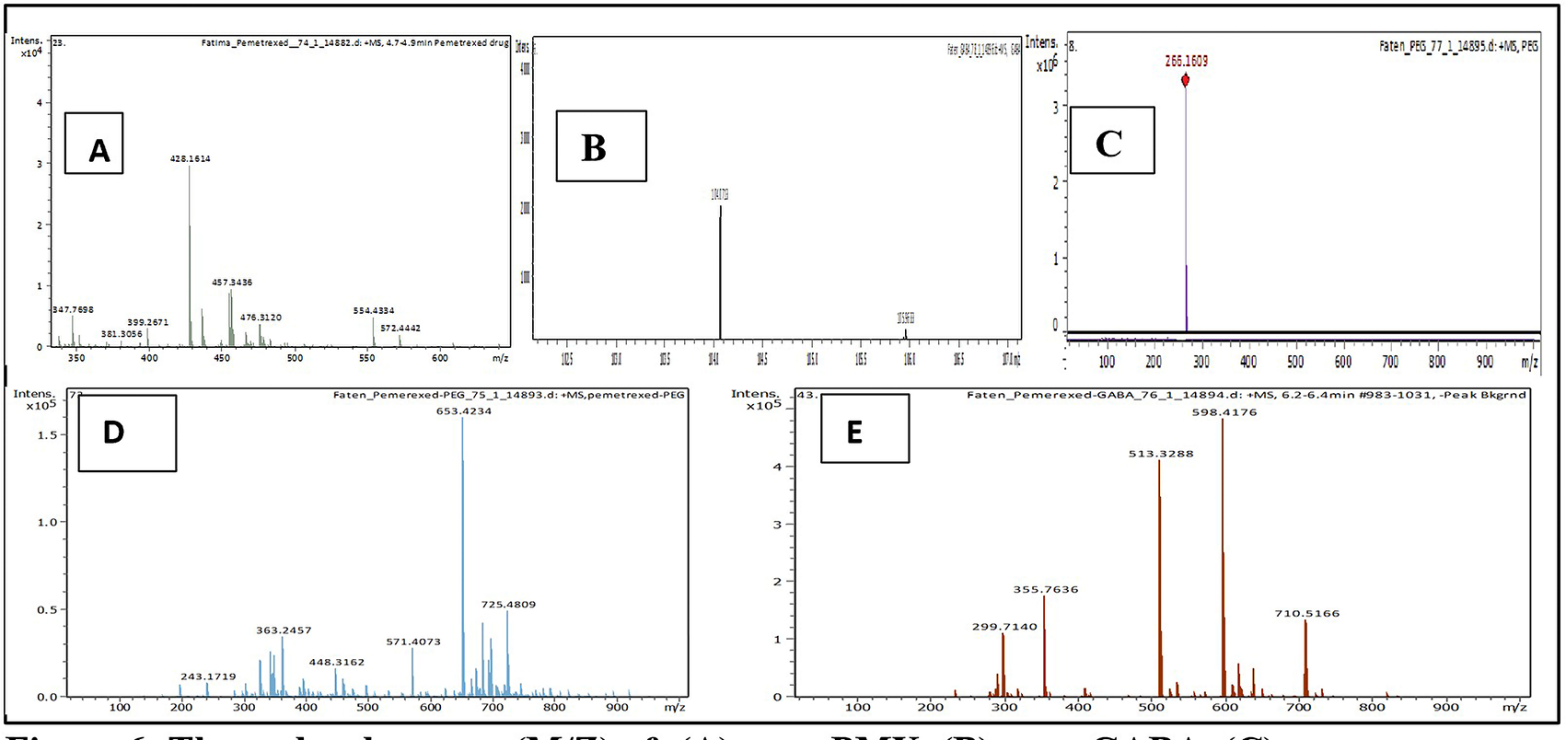
The molecular mass (M/Z) of: (A) pure PMX, (B) pure GABA, (C) pure PEG, (D) PMX-PEG linkage and (E) PMX-GABA linkage.

Mass divided by charge number (M/Z) represents the dividing mass number of ion by its charge, since the charge is almost equal to one, so the M/Z represents the molecular mass of the compound in the horizontal axis of chromatogram. ^40^ The (M/Z) for pure PMX 428.159, (M/Z) pure GABA 104.0713, (M/Z) pure PEG 266.16, the PMX-PEG linkage displayed different and multiple (M/Z) due to possibility of side reaction or unreacted materials. Among these multiple (M/Z) is 674, which may represent the intended molecular weight of our target molecule PMX-PEG linkage ( Figure 6). The final (M/Z) for PMX-GABA linkage displayed two main peaks 513.32 and 598.41, and it was concluded that the newly conjugated molecule may involve one GABA molecule conjugated by the α-COOH group of PMX to give the new entity with molecular weight 512 g/mol, while the second M/Z (598.4) may involve two molecules of GABA conjugated with the two α and γ COOH groups of PMX. Similar changes in M/Z in the LC-MS were observed in pegylated pemetrexed prodrug conjugated with amino acid linker. ^41^ The underlying data are available in a data repository. ^42^

### Characterization by ^1^HNMR

**Figure 7.**
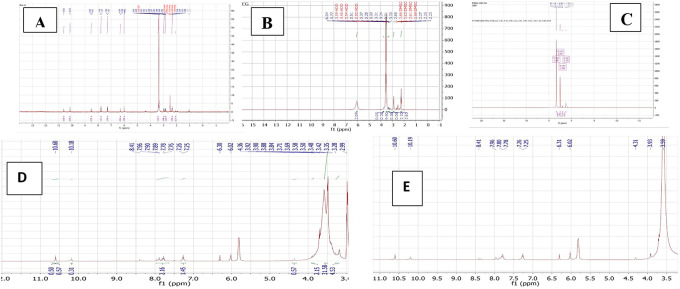
The ^1^HNMR spectrum of (A) PMX, (B) PEG, (C) GABA, (D) PMX-PEG linkage, and (E) PMX-GABA linkage.

The PMX-PEG linkage and PMX-GABA linkage were confirmed by ^1^HNMR spectra using DMSO as the solvent and all the results analyzed by MesterNova NMR analysis software and presented in Figure 7.

The ^1^HNMR spectrum of pure PMX gives a signal at 2.08–2.98, 3.31–3.41 ppm (−CH _2_−), 6.3 ppm (pyrrole −CH−), 7.2 and 7.7 ppm (aryl–CH), signal 10.15 ppm pteridine proton, 6.01 ppm aniline proton, and 10.62 ppm (-NH of pyrrole) and all agreed with reported values. ^43^

The typical ^1^HNMR spectrum of PEG gives signal 3.3–3.7 ppm for the protons of PEG chain, other signals in good agreement with reported data. ^44^

The ^1^HNMR spectrum of PMX-PEG linkage showed all the main characteristics peaks of pure PMX and showed all peaks presented with PEG linker with a slight shift in protons peaks that specifically involved in the linkage and amide bond formation. The ^1^HNMR spectrum for PMX-PEG linkage displaced the characteristics peaks for PEG after linkage at 2.86–3.9 ppm for the protons PEG chain, peaks spectrum of PMX after linkage showed all original PMX signal at 6.3 ppm (−CH− of pyrrole), 6.02 ppm for aniline protons of PMX, ^16^ 7.25 with 7.76 ppm (−CH aryl), 10.18 ppm for pteridine PMX proton, and 10.6 ppm (-NH of pyrrole). ^43^ The -CH _2_ of PMX neighboring to the α -COOH shifted from 2.33–2.36 to 2.26 ppm after linkage, also its neighboring -CH _2_ shifted from 2.08 to 1.7 ppm. The ^1^HNMR spectrum of PMX-PEG linkage displaced a peak at 3.35 ppm corresponding to -CH _2_ of PEG neighboring to terminal -NH _2_ after linkage and this was at 2.89 ppm for pure unreacted PEG, disappearance of the broad -NH _2_ PEG peak at 6.04 ppm and the appearance of a peak at 7.89 ppm in the PMX-PEG linkage ^1^HNMR spectrum, which corresponds to (-NH) proton of amide is further confirmed the intended linkage. ^45^

The ^1^HNMR spectrum of GABA displaced the characteristics GABA proton peaks at 1.23, 1.98, and 3.36 ppm (for −CH _2_−) as well as other peaks and all in good agreement with reported data. ^46^ The ^1^HNMR spectrum for PMX-GABA linkage showed all main peaks of PMX and GABA. The -CH _2_ proton peak of PMX adjacent to the α-COOH of PMX appeared as one peak range with the -CH _2_ proton peak neighboring to terminal -COOH of GABA in the PMX-GABA linkage, also the -CH _2_ proton peak of GABA neighboring to -NH shifted to 3.5 ppm (it was at 3.36 ppm in pure GABA) after linkage affected by adjacent amide bond, in addition to the appearance of new peak between 7.78–7.80 ppm in the ^1^HNMR spectrum of PMX-GABA linkage indicating the success of linkage and formation of amide bond. ^47^ Broadening of the -CH _2_ group of PMX in the ^1^HNMR spectrum, which is neighboring to the α- carboxyl group of PMX and appeared as broad peak at 3.93 ppm indicated reaction also occurred on the neighboring carbonyl group (conjugation of GABA with α-COOH of PMX). All other peaks appeared are correlated to PMX. These results of ^1^HNMR agreed with our results obtained with FTIR, DSC, and mass spectroscopy, which suggested two molecules of GABA linked by two amide bonds with two -COOH of PMX. The data of ^1^HNMR spectrum are available in a data repository. ^48^

### Characterization of pemetrexed-mAb conjugates

The successful linkage between PMX and each of PEG and GABA, proceeded by introducing the mAb (atezolizumab) to the prepared linkage to get two types of conjugates: PMX-PEG-AtZ mAb and PMX-GABA-AtZ mAb. Both conjugates were characterized.

### SDS-PAGE gel electrophoresis

**Figure 8.**
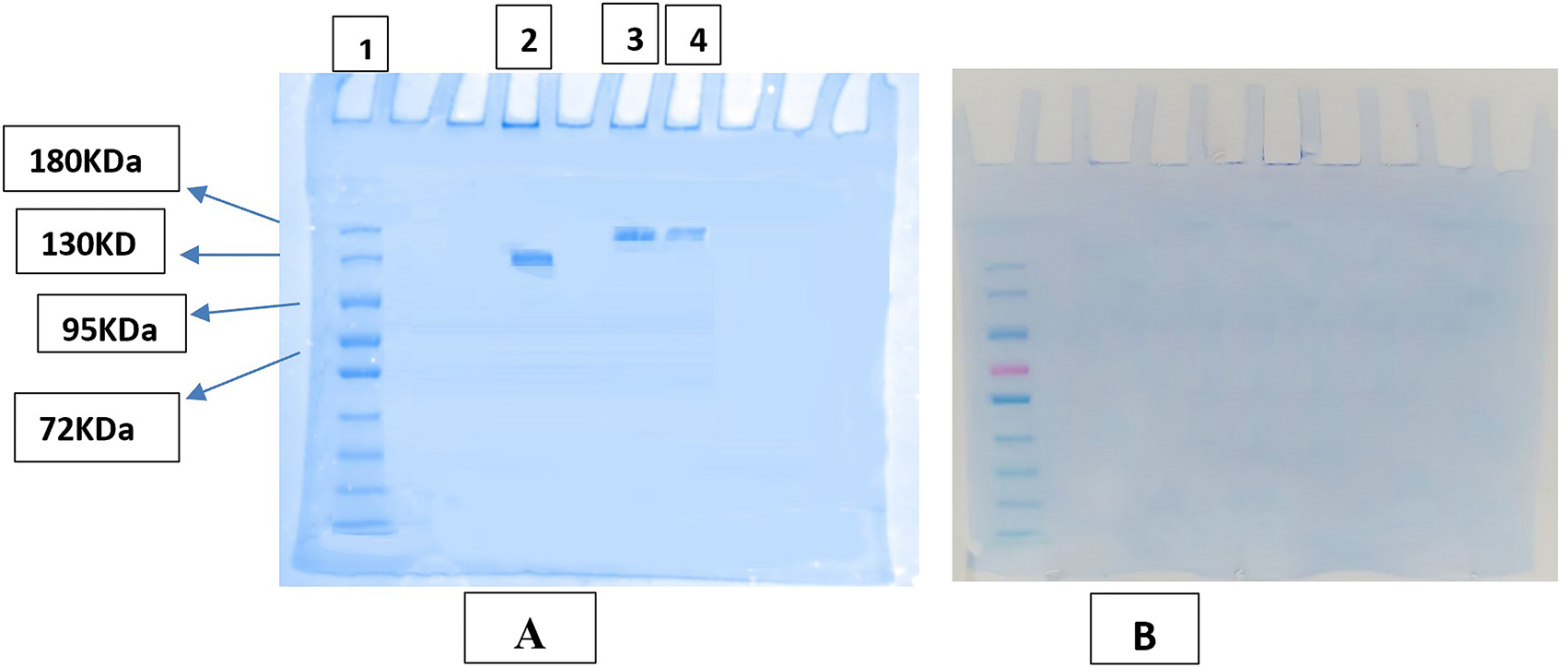
SDS-PAGE image of AtZ, PMX-AtZ conjugate. (A) samples without heating where (1): pre-stained protein marker, (2) AtZ mAb, (3) PMX-PEG-AtZ conjugate, and (4) PMX-GABA-AtZ conjugate. (B): heated samples of conjugate and unconjugated mAb.

Results of gel electrophoresis for samples without heating the samples (AtZ mAb, PMX-PEG-AtZ conjugate, and PMX-GABA-AtZ conjugate) showed in Figure 8 (A) in which mAb showed band near 130 KDa and the two conjugates (PMX-PEG-AtZ and PMX-GABA-AtZ) showed band at 180 KDa and this confirmed the conjugation and the mAb was not affected by the experimental condition during conjugation. The same samples (unconjugated mAb and the two conjugates), which were heated 95°C for 5 minutes before being introduced into the wells of assembled gel (which is the common method applied for many proteins in PAGE ^49^) showed no bands indicating unfolding of mAb by heating ( Figure 8B), as atezolizumab had limited stability for only few hours at 20-25°C. ^50^ These results were in agreement with other study in which antibody conjugated with maytansinoid and auristatin demonstrated that both unconjugated and conjugated antibody were unfolded properly by heating to 95°C. ^51^ The data are available in a data repository. ^52^

### LC-MS

**Figure 9.**
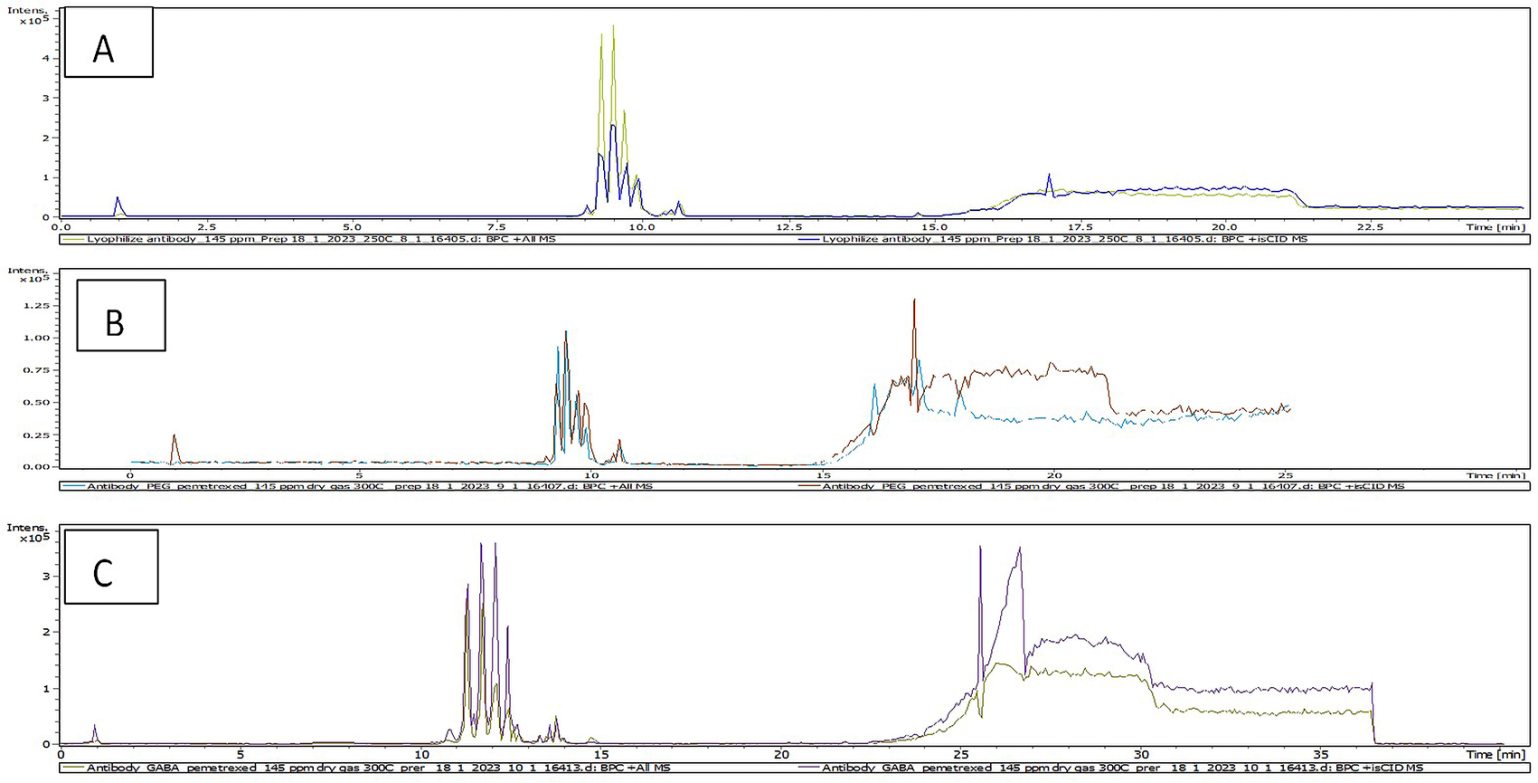
Total ion chromatogram (TIC) for (A) pure AtZ mAb, (B) PMX-PEG-AtZ conjugate, and (C) PMX-GABA-AtZ conjugate.

**Figure 10.**
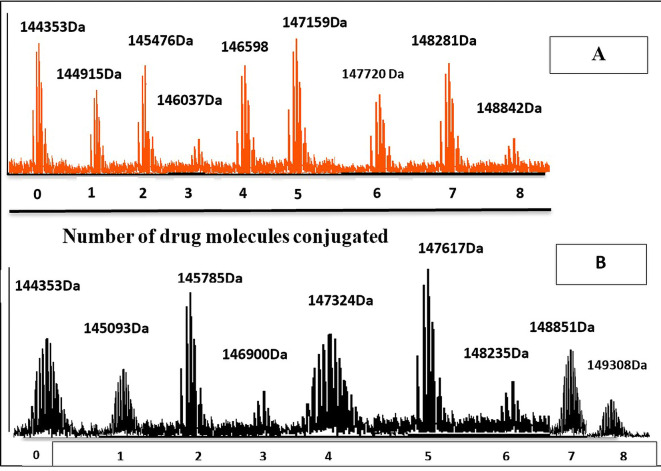
The molecular mass of: (A) PMX-GABA-AtZ conjugate, and (B) PMX-PEG-AtZ conjugate.

The LC-MS is beneficial tool for characterization of antibody and identification of antibody conjugate by detection of antibody mass. ^53^ The total ion chromatogram (TIC) results of characterization of AtZ mAb, PMX-PEG-AtZ conjugate, and PMX-GABA-AtZ conjugate are shown in Figure 9 and revealed a change in chromatograms of conjugate in comparison to unconjugated pure antibody and represented the major conjugated molecules eluted after 15 minutes. These results are in accordance with another study in which trastuzumab conjugated to microtubule disrupting agent. ^54^ The mass profile of AtZ-PMX conjugate were simplified ( Figure 10) with only m/z values (results showed up to eight molecules of the PMX-PEG linkage and PMX-GABA linkage were conjugated to AtZ with increase in molecular mass of antibody), these results in accordance with Trastuzumab deruxtecan conjugate in which mAb conjugated 8 deruxtecan molecules through a maleimide based linker and gain approval for breast cancer. ^55^ The drug: antibody ratio calculated as the average PMX linkage conjugated based on the sum of all intensities. ^56^ The calculated drug: antibody ratio of PMX-AtZ conjugate using two types of linker PEG and GABA was 4 and 3.74, respectively. The obtained PMX-AtZ conjugation ratio agreed with that Ado-trastuzumab emtansine (first ADC approved) for malignancy in which emtansine conjugation with trastuzumab in average of 3 to 4 (emtansine molecules): 1 (mAb molecule) using stable thioether linker. ^57^ The data are available in a data repository. ^58^

**Figure 11.**
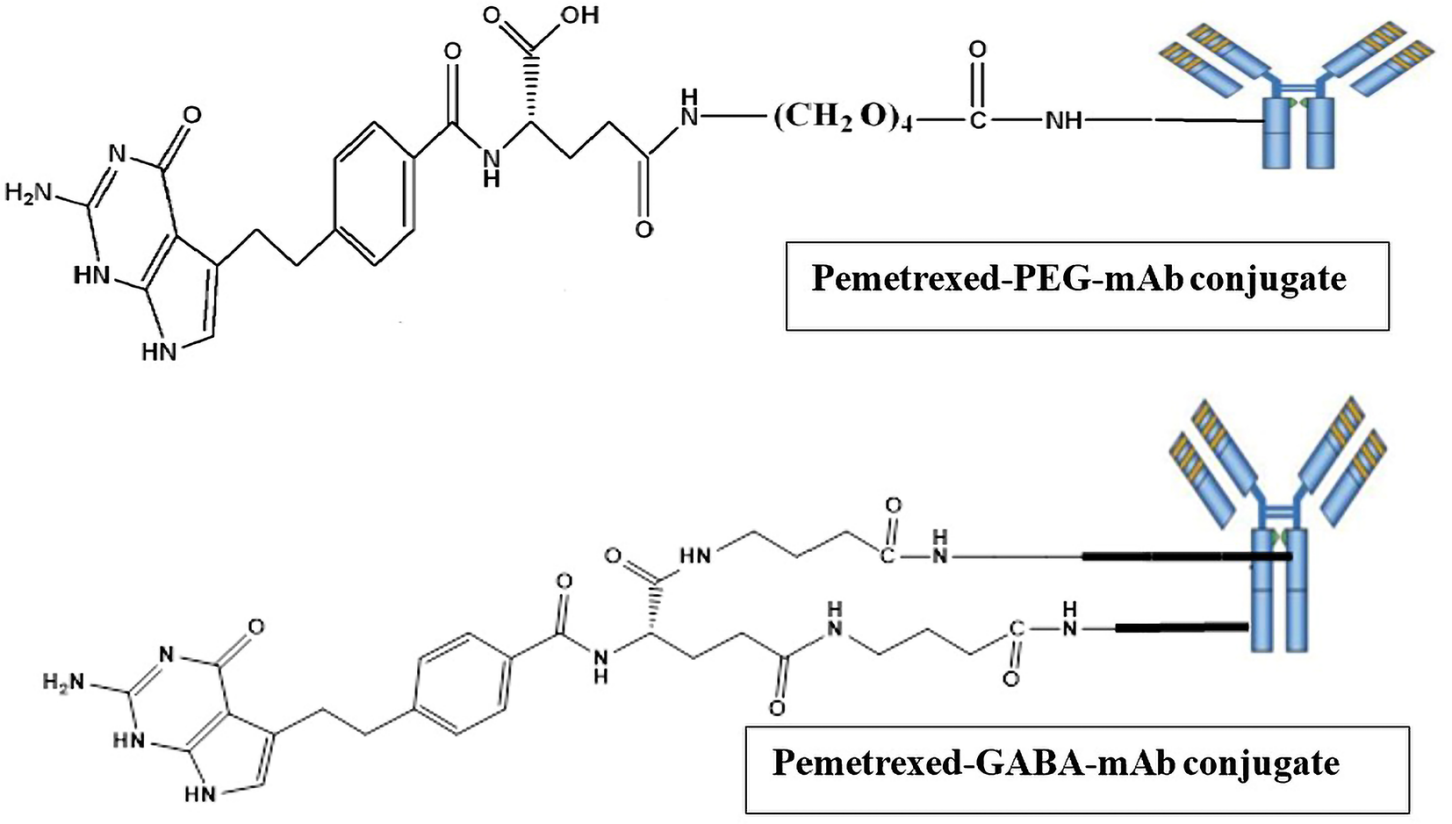
Expected structures of the PMX-AtZ mAb conjugate using PEG and GABA each one separately as linker. This figure is an original figure produced by the authors for this article.

In accordance with data obtained from the applied characterizations, the expected structure for 1:1 PMX-AtZ mAb conjugation is suggested in Figure 11, where in PMX-PEG-AtZ mAb conjugate one molecule of PMX linked to one molecule of PEG, which in turn linked with one molecule of mAb. While 1:1 of PMX-GABA-AtZ mAb conjugation, where one molecule of PMX linked to two molecules of GABA, which in turn conjugate with one molecule of mAb.

### Cytotoxic activity study for the prepared conjugates

Cells treated with increasing concentrations of AtZ mAb, pure PMX, PMX-PEG linkage, PMX-GABA linkage, PMX-PEG-AtZ mAb conjugate, and PMX-GABA-AtZ mAb conjugate.

**Figure 12.**
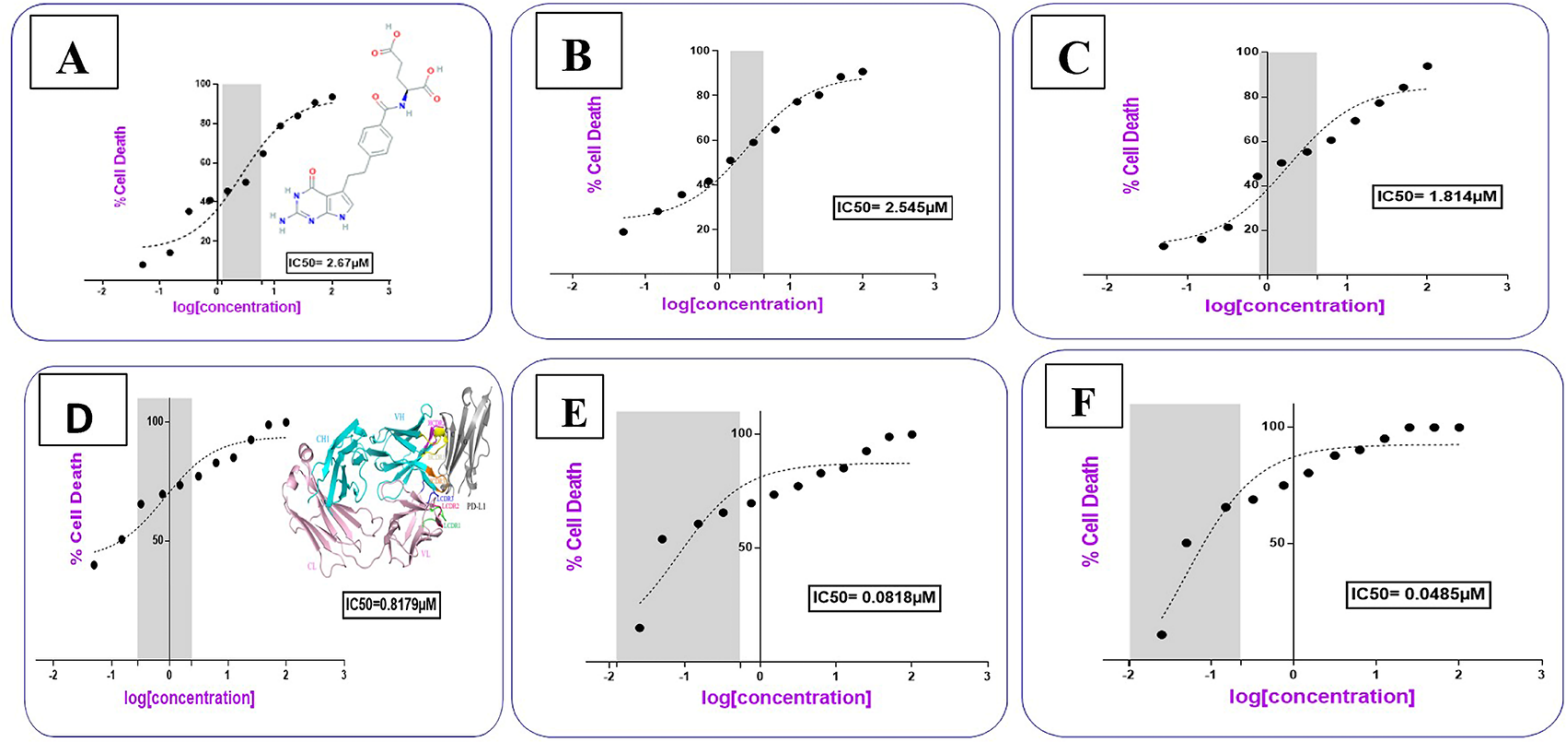
IC _50_ of (A) pure PMX, (B) PMX-PEG linkage, and (C) PMX-GABA linkage, (D) pure AtZ mAb, (E) PMX-PEG-AtZ mAb conjugate, and (F) PMX-GABA-AtZ mAb conjugate.

The PMX-PEG linkage and PMX-GABA linkage showed lower IC _50_ (2.54, 1.814 μM, respectively) than that of pure PMX (2.67 μM) on cancer cell at different time (24, 48 and 72 h) ( Figure 12). These results indicated that PMX-PEG linkage could potentiate drug cytotoxic activity due to enhancement of solubility and therefore enhances cell uptake and penetration, and this in agreement with other study demonstrated that the incorporation of PEG altered paclitaxel delivery mechanism and enhanced cytotoxic efficacy. ^59^ The IC _50_ (1.814 μM) obtained with PMX-GABA linkage was significantly (P≤ 0.05) lower than pure PMX and PMX-PEG linkage due to the synergistic effect of conjugation, where a previous reported data assumed that GABA had a strong inhibitory effect on cell proliferation. ^60^ In addition, the gene expression of GABA receptor is high in lung cancer and the proliferation index for such cancer is inversely correlated with GABA concentration when given exogenously, therefore GABA inhibits proliferation of the lung cancer in time and dose manner. ^61^

AtZ mAb gave IC _50_ 0.817 μM ( Table 1), which is similar to reported data, ^21^ indicating that the lyophilized mAb kept its anticancer activity. The complete conjugation (PMX-PEG-AtZ mAb) and (PMX-GABA-AtZ mAb) showed potent cytotoxic effect where the IC _50_ was 0.0818 and 0.0485 μM for each, respectively ( Figure 12). The two types of conjugation were significantly (P≤ 0.05) more cytotoxic activity than PMX alone and antibody alone, which demonstrated that hybrid effect of the final conjugates significantly enhanced cytotoxicity due to synergistic effect. The data are available in a data repository. ^62^ Original data by Graph Pad Prism are available in a data repository in a pzfx and JPG format. ^63^

**Table 1.** IC _50_ Values for the prepared conjugates in comparison to their pure components and linkages.

Compound name	IC _50_ (μM)
Pemetrexed	2.67
Pemetrexed-PEG linkage	2.54
Pemetrexed-GABA linkage	1.814
Atezolizumab (mAb)	0.817
Pemetrexed-PEG-mAb conjugate	0.0818
Pemetrexed-GABA-mAb conjugate	0.0485

It was noted that using GABA (as a linker) for PMX-AtZ mAb conjugation gave higher cytotoxic effect than using PEG as a linker, which could be attributed to the involvement of two molecules of GABA in the PMX-AtZ mAb conjugation that may cause further enhancement (≈ 2 folds) in cytotoxic effect of the final conjugate. According to our knowledge no previous work was reported on using GABA as a linker for drug-monoclonal antibody conjugation.

**Figure 13.**
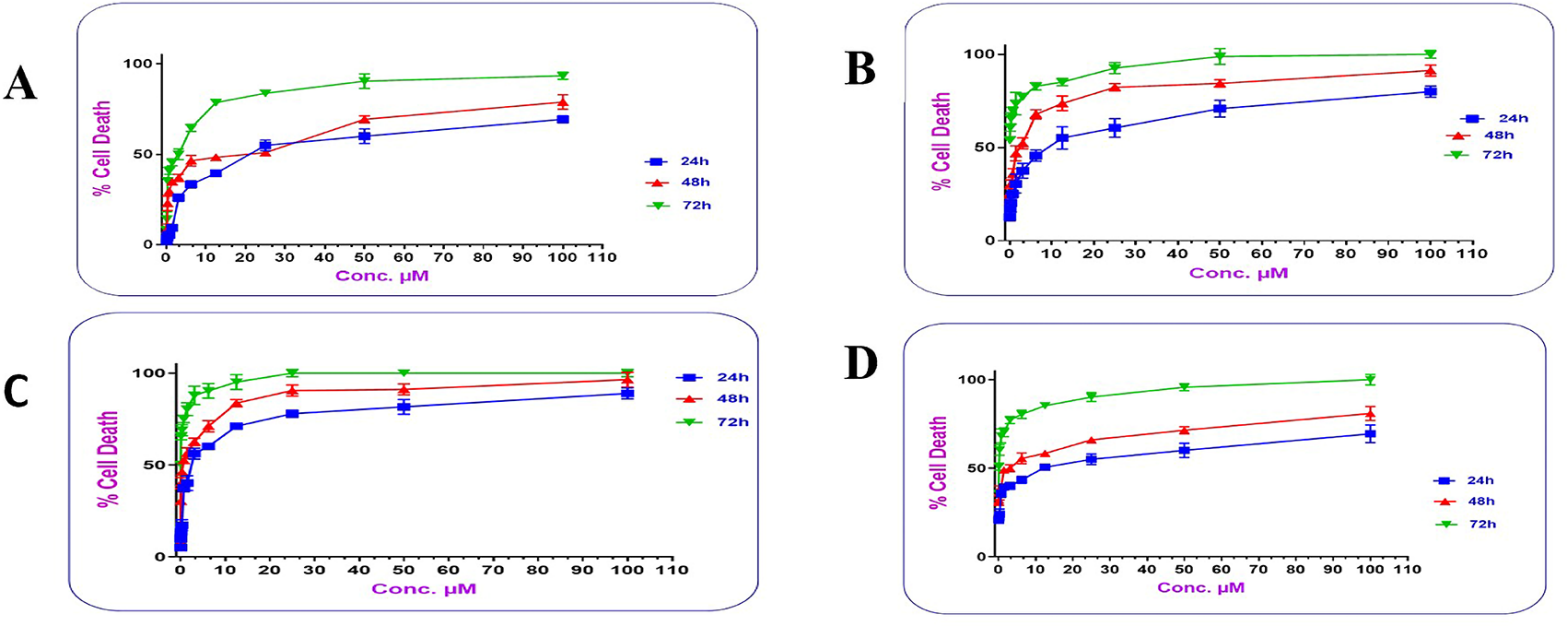
The dose response curve for different concentrations in lung cancer cell of (A) PMX, (B) PMX-PEG-AtZ mAb conjugate, (C) PMX-GABA-AtZ mAb conjugate, and (D) AtZ mAb.

The dose-response curve showed that the cytotoxic activity of the two types of the prepared conjugates was dose and time dependent. Figure 13 shows that as the concentrations of the conjugates increased; their cytotoxic activity increased significantly (P≤ 0.05), where at concentration 12.5 μM; PMX-GABA-AtZ mAb and PMX-PEG-AtZ mAb conjugates gave 71.168% and 55.167% cell death, respectively, after 24 h in comparison to pure PMX, which gave 39.58% cell death and the cell death continued to reach 88.987% and 80% for the two conjugates as well as 69.43% for pure PMX at 100 μM concentration after 24 h and similar increase in response upon increasing concentration was observed after 48 and 72 h. This suggests that the higher percentage of cell death achieved by the two PMX-AtZ mAb conjugates attributed to the collaborative effect of PMX and AtZ mAb upon conjugation. The enhancement of cytotoxic activity of drug-mAb conjugation observed in this study agreed with reported enhancement observed upon conjugation of auristatin with trastuzumab and attributed to the synergistic effect of both the drug and antibody. ^64^

In this study, it was observed that in all the working concentrations; the PMX-GABA-AtZ mAb conjugate showed a significantly (P≤ 0.05) higher anticancer effect against the lung cancer cells indicating the contribution of the inhibitory effect of GABA on these cancer cells. ^65^ The data are available in a data repository in a pzfx and PDF format. ^66^

**Figure 14.**
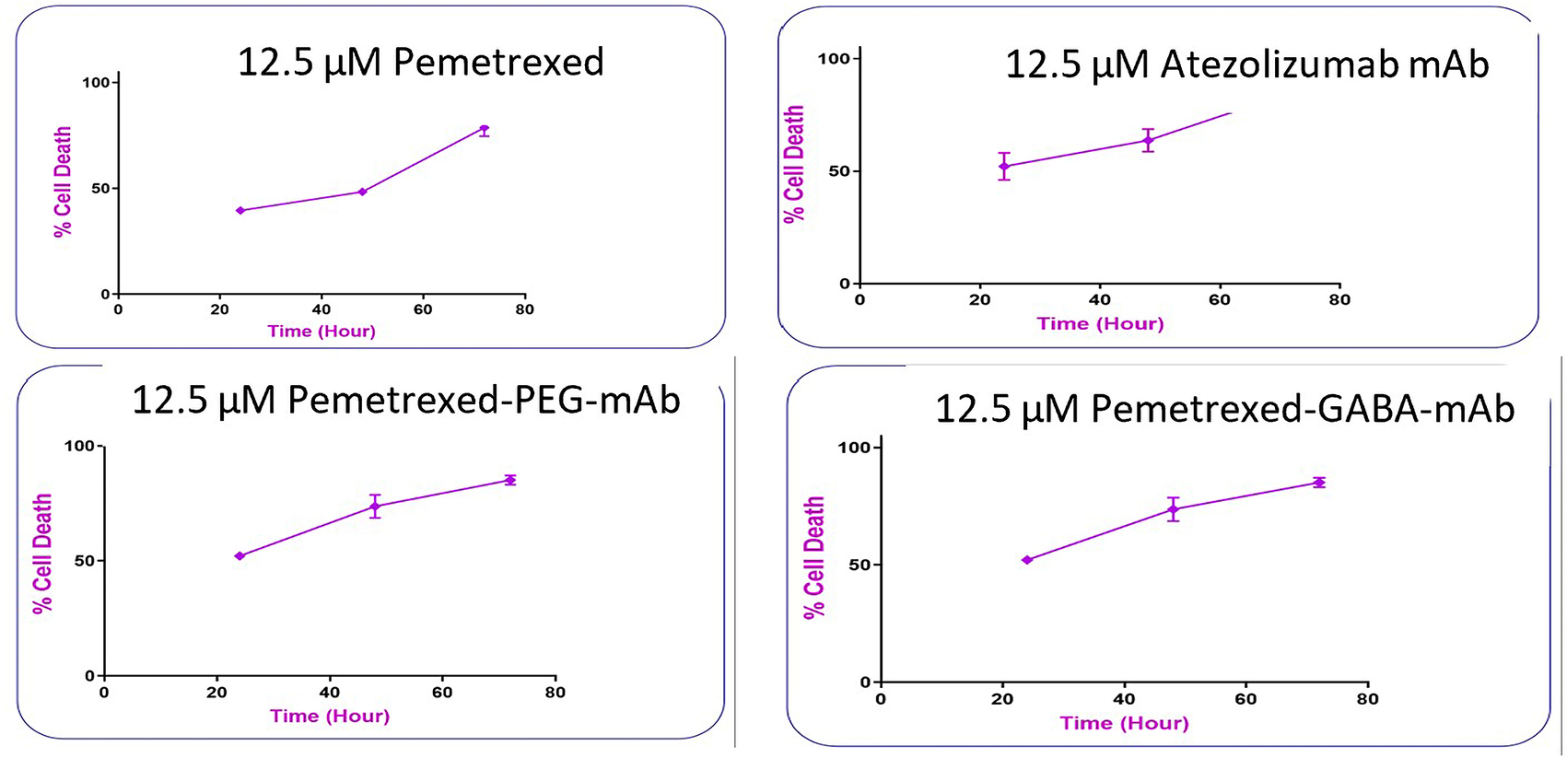
The time response curve at different times in lung cancer cells.

The time response curve ( Figure 14) shows that with 12.5 μM of pure PMX at 24 h gave (39.58% cell death), at 48 h (48.39% cell death) and 72 h (78.70% cell death) indicating the drug activity is time dependent, which agreed with reported data for PMX. ^29^^,^^67^

AtZ mAb with 12.5 μM concentration at 24 h lead to (52% cell death), at 48 h (63% cell death) and 72 h (85.14% cell death). which was also time dependent and agreed with reported data for AtZ, ^68^ but it was significantly (P≤ 0.05) higher than PMX confirming the potency (as well as selectivity) of the mAb as anticancer agent. ^69^

The PMX-PEG-AtZ mAb conjugate showed also time response and higher cytotoxic effect than PMX where it gave (with concentration 12.5 μM) cell death 52.16% at 24 h, 73.69% at 48 h, and 85.14% at 72 h, but it was not significantly higher than pure mAb.

The PMX-GABA-AtZ mAb conjugate (12.5 μM concentration) showed significant superior high percent of cell death with time than pure drug and PMX-PEG-AtZ mAb conjugate where it gave 71.16% cell death after 24 h, 83.69% after 48 h, and 95.17% cell death after 72 h. This confirms the contribution of GABA in enhancing the anticancer activity of both PMX and AtZ mAb in dose and time dependent manner. The data are available in a data repository in a pzfx and PDF format. ^70^

## Conclusions

This work succeeded to prepare pemetrexed-atezolizumab conjugation (for the first time) using two types of linkers PEG and GABA (each one separately) in an average ratio of 4 (pemetrexed molecules):1 (atezolizumab mAb) with significantly higher cytotoxic effects than each of pemetrexed and mAb alone indicating the potential use of such conjugates in a low dose leading to reduction of the serious side effect of pemetrexed that may be selectively delivered towards the lung cells where antigen is overexpressed as well as to reduction in the amount and cost of therapeutically mAb used. This work also revealed the efficacy of linkers used for such conjugation namely GABA, which was newly used in this work for drug-mAb conjugation where two molecules of GABA contributed and resulted in significantly higher cytotoxic effects compared to PEG (the commonly used linker in many drug-mAb conjugation). This may confirm that GABA molecule possesses anticancer effect which resulted in synergistic anticancer effect in the prepared pemetrexed-GABA-atezolizumab conjugate.

## Data Availability

Zenodo: Figures of FTIR. https://doi.org/10.5281/zenodo.8132774. ^36^ This project contains the following underlying data:
- FTIR figures (pdf ). Zenodo: Figures of DSC. https://doi.org/10.5281/zenodo.8132703. ^39^ This project contains the following underlying data:
- DSC figures (jpg). Zenodo: Data for LC-Mass. https://doi.org/10.5281/zenodo.8132322. ^42^ This project contains the following underlying data:
- Excel files contain figures for pemetrexed, PEG, GABA, pemetrexed-PEG conjugate, and pemetrexed-GABA conjugate represented the retention time for each one and molecular mass detection for each compound. Zenodo: Figures of 1HNMR. https://doi.org/10.5281/zenodo.8180439. ^48^ This project contains the following underlying data:
-
^1^HNMR figures (pdf ) for pemetrexed. PEG, GABA, pemetrexed-PEG conjugate, and pemetrexed-GABA conjugate. Zenodo: Figure of gel electrophoresis. https://doi.org/10.5281/zenodo.8132813. ^52^ This project contains the following underlying data:
- Gel electrophoresis figure (tif ). Zenodo: Data of LC-Mass. https://doi.org/10.5281/zenodo.8132226. ^58^ This project contains the following underlying data:
- Excel file for unconjugated antibody (atezolizumab) - Excel file for pemetrexed-PEG-atezolizumab conjugate - Excel file for pemetrexed-GABA-atezolizumab conjugate Zenodo: Excel data for MTT assay by promega. https://doi.org/10.5281/zenodo.8132166. ^62^ This project contains the following underlying data:
- Excel data for MTT assay Zenodo: Data of IC _50_. https://doi.org/10.5281/zenodo.8243096. ^63^ This project contains the following underlying data:
- IC50 data by Graph pad prism in a pzfx format. - IC50 data in a JPG format. Zenodo: Figures of Dose response curve. https://doi.org/10.5281/zenodo.8243127. ^66^ This project contains the following underlying data:
-
Figures of Dose Response Curve by Grap Pad Prism in a pzfx format. -
Figures of Dose Response Curve in a PDF format. Zenodo: Figures of Time response curve. https://doi.org/10.5281/zenodo.8243151. ^70^ This project contains the following underlying data:
- Time response curve figures by Grap Pad Prism in a pzfx format. - Time response curve figures in a PDF format. Data are available under the terms of the Creative Commons Attribution 4.0 International license (CC-BY 4.0).
