# Supplementary material for: Prospective effect of linkers type on the anticancer activity of pemetrexed-monoclonal antibody (atezolizumab) conjugates
Source: F1000Res. 2024 Mar 11;12:1197. Originally published 2023 Sep 25. [Version 2] doi: 10.12688/f1000research.140284.2 (PMC11320184; doi:10.12688/f1000research.140284.2)
Supplement: Peer review history

# Reviewer report: Reviewer response for version 2

**DOI:** 10.5256/f1000research.163486.r309198

**Published:** 2024-08-12

**Authors:** Youlin Zeng

**Affiliations:** 1 Hunan Normal University, Changsha, Hunan, China

This work introduced the synthesis of atezolizumab-pemetrexed conjugate using two types of linkers (linker conjugated with -NH2 of lysine amino acid in the mAb) for the  anticancer activity against lung cells A549. However, there are still some minor issues in the article that need to be corrected. The reviewer believes this work can be indexed after addressing the following minor revisions.

 1) Page 4, sentence “Hydroxy -2, 5-dioxopyrrolidine-3-sulfonicacid sodium salt (Sulfo-NHS)”, please delete the space between 2, and 5.

 2) Page 6, please change “5 x 10 ^3^” to “5 × 10 ^3^”. Same changes are needed for others.

 Fig. 2, scale and unify the box which covered the label of vertical axis. Also, please unify the box in Fig. 3.

 3) Fig. 5, why PMX-GABA linkage have two retention times (5.2 and 6.2 min)? not a pure compound? Which retention time correspond to the PMX bonded one GABA? Which retention time correspond to the PMX bonded two GABA? Can purify the mixture or not? Can get the pure PMX- *one*-GABA-mAb and PMX- *two*-GABA-mAb? If can do, and obtain the corresponding IC _50_, which may prove the conclusion (It was noted that using GABA (as a linker) for PMX-AtZ mAb conjugation gave higher cytotoxic effect than using PEG as a linker) listed page 13, the last but one paragraph.

Is the work clearly and accurately presented and does it cite the current literature?

Yes

If applicable, is the statistical analysis and its interpretation appropriate?

Yes

Are all the source data underlying the results available to ensure full reproducibility?

Yes

Is the study design appropriate and is the work technically sound?

Yes

Are the conclusions drawn adequately supported by the results?

Partly

Are sufficient details of methods and analysis provided to allow replication by others?

Yes

Reviewer Expertise:

Design and assay of antitumor drugs

I confirm that I have read this submission and believe that I have an appropriate level of expertise to confirm that it is of an acceptable scientific standard.

# Reviewer report: Reviewer response for version 1

**DOI:** 10.5256/f1000research.153622.r247798

**Published:** 2024-03-01

**Authors:** Khalid Kadhem Al-Kinani

**Affiliations:** 1 University of Baghdad, Baghdad, Baghdad Governorate, Iraq

- Extensive amount of work and a very well written manuscript.

 -  It would be better to make clearer that DSC analyses were done for the "chemical parts' of the ADC but not the entire ADC (AtZ-PMX). Future studies could include DSC for the prepared ADC from stability point of view.

 - It might be better to delete table 1 since SDS-PAGE components are widely used and can be found very easily elsewhere. Instead, the authors can focus on the other interesting findings of this work which are plenty, according to my opinion.

 - UHPLC is not Mass spectroscopy. So, correct that in the methods section (Mass spectroscopy) accordingly and mention the full LC-MS setup.

 -  SDS_PAGE gel, the experimental section needs to provide better description in terms of heated and non-heated samples to better explain panel B of Figure 8. Also, the statement " atezolizumab had limited stability for only few hours at 20-25 C" could not be very true since mAbs are fairly stable for many days if not months.

Is the work clearly and accurately presented and does it cite the current literature?

Yes

If applicable, is the statistical analysis and its interpretation appropriate?

Yes

Are all the source data underlying the results available to ensure full reproducibility?

Partly

Is the study design appropriate and is the work technically sound?

Yes

Are the conclusions drawn adequately supported by the results?

Yes

Are sufficient details of methods and analysis provided to allow replication by others?

Yes

Reviewer Expertise:

Pharmaceutical nanotechnology and biotechnology including monoclonal antibody and antibody drug conjugates.

I confirm that I have read this submission and believe that I have an appropriate level of expertise to confirm that it is of an acceptable scientific standard, however I have significant reservations, as outlined above.

# Response

**Published:** 2024-03-06

**Authors:** faten ibraheem

**Affiliations:** pharmaceutics, Mustansiriyah University, Baghdad, Baghdad Governorate, Iraq

Thanks Dr. Khalid for your valuable review comments.

 We are going forward in our future research to discover more about Atezolizumab stability and specifically temprature effect.

 We appreciate your feedback.

# Reviewer report: Reviewer response for version 1

**DOI:** 10.5256/f1000research.153622.r216712

**Published:** 2023-10-23

**Authors:** Ghazi Al Jabal

**Affiliations:** 1 Universiti Sains Malaysia, Gelugor, Penang, Malaysia

This research article explores the potential impact of different linkers on the anticancer activity of a pemetrexed-monoclonal antibody conjugate. The study successfully demonstrates the conjugation of pemetrexed (PMX) and atezolizumab (AtZ) using two distinct linkers: polyethylene glycol (PEG) and gamma-aminobutyric acid (GABA). Various analytical techniques were employed to evaluate the PMX-PEG and PMX-GABA conjugates with AtZ. Notably, the PMX-GABA-AtZ conjugate exhibited enhanced cytotoxic activity when compared to pure PMX, AtZ, and the PMX-PEG-AtZ conjugate. The study suggests that such a conjugate has the potential to reduce the side effects and costs associated with pemetrexed chemotherapy and therapeutic monoclonal antibodies.

Is the work clearly and accurately presented and does it cite the current literature?

Yes

If applicable, is the statistical analysis and its interpretation appropriate?

Not applicable

Are all the source data underlying the results available to ensure full reproducibility?

Yes

Is the study design appropriate and is the work technically sound?

Yes

Are the conclusions drawn adequately supported by the results?

Yes

Are sufficient details of methods and analysis provided to allow replication by others?

Yes

Reviewer Expertise:

Drug design, Analytical analysis

I confirm that I have read this submission and believe that I have an appropriate level of expertise to confirm that it is of an acceptable scientific standard.

# Response

**Published:** 2024-03-06

**Authors:** faten ibraheem

**Affiliations:** pharmaceutics, Mustansiriyah University, Baghdad, Baghdad Governorate, Iraq

Thanks Dr. Ghazi for your valuable reviewing
